# Effects of Mesenchymal Stem Cells on Functions of Chimeric Antigen Receptor-Expressing T Lymphocytes and Natural Killer Cells

**DOI:** 10.3390/cells14130978

**Published:** 2025-06-25

**Authors:** Vladislav Volarevic, Carl Randall Harrell, Aleksandar Arsenijevic, Valentin Djonov, Ana Volarevic

**Affiliations:** 1 Departments of Genetics, Microbiology and Immunology, Center for Research on Harmful Effects of Biological and Chemical Hazards, Faculty of Medical Sciences, University of Kragujevac, 69 Svetozara Markovica Street, 34000 Kragujevac, Serbia; aleksandar@medf.kg.ac.rs; 2 Regenerative Processing Plant, LLC, 34176 US Highway 19 N, Palm Harbor, FL 34684, USA; 3 Institute of Anatomy, University of Bern, Baltzerstrasse 2, 3012 Bern, Switzerland; valentin.djonov@unibe.ch; 4 Departments of Psychology, Center for Research on Harmful Effects of Biological and Chemical Hazards, Faculty of Medical Sciences, University of Kragujevac, 69 Svetozara Markovica Street, 34000 Kragujevac, Serbia; ana.volarevic@fmn.kg.ac.rs

**Keywords:** mesenchymal stem cells, chimeric antigen receptor, immune cells, immunotherapy, malignant diseases

## Abstract

Chimeric antigen receptor (CAR)-engineered immune cells, particularly CAR T lymphocytes and CAR natural killer (NK) cells, have revolutionized cancer immunotherapy. However, their therapeutic efficacy and safety can be influenced by the tumor microenvironment, particularly the presence of mesenchymal stem cells (MSCs). MSCs are immunomodulatory cells which can alter the function of tumor-infiltrated immune cells in both supportive and suppressive ways. Results obtained in recently conducted experimental studies demonstrate that MSCs modulate proliferation, cytotoxicity, cytokine production and anti-tumor activity in CAR-expressing immune cells in both a juxtacrine and a paracrine manner. While MSCs can enhance CAR cell viability and persistence through trophic support, they may also impair cytotoxic function and promote an immunosuppressive phenotype under certain conditions. Understanding the dualistic nature of MSCs in CAR-based immunotherapy for malignant diseases is critical for optimizing clinical outcomes. Additionally, MSCs may serve as vehicles for targeted delivery of immunomodulatory agents, and should be considered as active components in the design of next-generation CAR-based immunotherapies. Accordingly, in this review article we emphasize molecular and cellular mechanisms involved in MSC-dependent modulation of CAR-expressing immune cells, paving the way for more efficient CAR-based immunotherapy for malignant diseases.

## 1. Introduction

The concept of the Chimeric Antigen Receptor (CAR) was first introduced in 1987 by Kuwana and colleagues, who constructed chimeric receptors by fusing immunoglobulin-derived variable regions with T-cell receptor (TCR)-derived constant regions [1]. These chimeric receptors were expressed in murine T-cell lymphoma EL4 cells. Upon exposure to phosphorylcholine, a component of the bacterial cell wall, the transfected T cells exhibited calcium influx, indicating activation independent of the major histocompatibility complex (MHC) [1]. This foundational study established the conceptual and experimental basis for CAR-based immunotherapy, a paradigm-shifting advancement in oncology that has enabled precise, antigen-specific targeting of malignant cells [1,2,3,4]. This innovative approach involves the genetic modification of a patient’s own immune cells to express synthetic receptors designed to recognize tumor-associated antigens presented on the surface of tumor cells [1]. These engineered receptors combine the antigen-binding properties of antibodies with the cytotoxic functions of T cells, enabling a more direct and effective immune response against cancer [1]. By enhancing the precision and potency of immune cell activity, this approach offers a novel and promising therapeutic strategy, particularly for hematological malignancies, and represents a significant shift from conventional treatments such as chemotherapy or radiation therapy [1,2]. By redirecting immune cells to recognize and destroy cancer cells in a MHC-independent manner, CAR-based immunotherapies have demonstrated remarkable efficacy in clinical settings, particularly for the treatment of hematological malignancies such as B-cell acute lymphoblastic leukemia (B-ALL), diffuse large B-cell lymphoma (DLBCL), and multiple myeloma [5,6,7]. The most well-established successes involve CAR T cells targeting CD19, with clinical trials reporting complete remission rates of up to 90% in pediatric and young-adult patients with relapsed or refractory B-ALL [5]. Similarly, CAR-based immunotherapy has demonstrated substantial therapeutic efficacy in the treatment of DLBCL, with CD19-targeted CAR T-cell therapies achieving high overall response rates and durable remissions in patients with relapsed or refractory disease who have limited treatment options [6]. In therapy for multiple myeloma, B-cell maturation antigen (BCMA)-targeted CAR T products have shown high response rates in CAR T-treated patients [7].

Despite these achievements, the efficacy of CAR T-cell therapy in solid tumors remains limited [2,3]. Challenges such as poor trafficking and infiltration into tumor sites, antigen heterogeneity, and the presence of immunosuppressive cellular components in the tumor microenvironment have hindered sustained responses [3]. Additionally, CAR-based immunotherapy faces several important limitations which have to be addressed [2,3]. One of the primary challenges is antigen escape, where tumor cells down-regulate or lose expression of the target antigen, rendering the CAR-modified cells ineffective [2,3]. There is also the risk of on-target, off-tumor effects, as when normal tissues expressing low levels of the target antigen are inadvertently attacked, leading to organ damage [2]. Moreover, the immunosuppressive tumor microenvironment, particularly in solid tumors, can inhibit CAR cell function and survival [3].

There are several serious adverse effects associated with CAR-based immunotherapy [8]. Cytokine Release Syndrome (CRS) and Immune Effector Cell-Associated Neurotoxicity Syndrome (ICANS) are potentially life-threatening side effects of CAR-based immunotherapy [8,9,10]. CRS is a systemic inflammatory response triggered by the rapid activation and proliferation of CAR-modified immune cells upon encountering their target antigen [9]. This activation leads to a massive release of pro-inflammatory cytokines such as interleukin (IL)-6, interferon gamma (IFN-γ), and tumor necrosis factor alpha (TNF-α), resulting in symptoms that range from mild fever and fatigue to severe hypotension, hypoxia, and multi-organ failure [9]. ICANS, on the other hand, is characterized by a spectrum of neurological symptoms, including confusion, aphasia, seizures, and, in severe cases, cerebral edema and coma [10]. While the precise mechanisms are not fully understood, ICANS is believed to result from cytokine-mediated endothelial activation and increased blood–brain barrier permeability, allowing neurotoxic factors to affect the central nervous system [10].

Mesenchymal stem cells (MSCs) are multipotent stromal cells capable of differentiating into various mesenchymal lineages, including osteoblasts, chondrocytes, and adipocytes [11,12,13]. One of the most important and clinically relevant properties of MSCs is their ability to modulate immune responses, both through direct cell–cell contact and by secretion of various bioactive molecules [12,13]. However, it is increasingly recognized that MSCs are not a uniform cell type, but rather a heterogeneous population with diverse phenotypic and functional characteristics influenced by their tissue of origin and the local microenvironment [11]. MSCs were initially identified in the bone marrow [11]. Bone marrow-derived MSCs (BM-MSCs) possess robust osteogenic potential and have been shown to exert strong immunosuppressive effects, making them attractive candidates for therapies targeting autoimmune and inflammatory diseases [11,12]. However, harvesting bone marrow is invasive, and the yield of MSCs is relatively low [11]. As a result, alternative tissue sources have been explored, including adipose tissue, umbilical cord, placenta, dental pulp, synovial membrane, and amniotic fluid [11]. Adipose tissue-derived MSCs (AT-MSCs) are relatively easy to obtain in large numbers and have shown a strong capacity for adipogenic differentiation [11]. They exhibit potent immunomodulatory functions, often comparable to or exceeding those of BM-MSCs, though their effects can be more variable depending on donor characteristics such as age and body mass index [11,12]. Umbilical cord-derived MSCs (UC-MSCs), particularly from Wharton’s jelly, are neonatal in origin and thus considered more “primitive” [11]. They exhibit high proliferative capacity and are thought to possess stronger immunosuppressive potential due to their low immunogenicity and enhanced secretion of anti-inflammatory cytokines TGF-β and IL-10 [11,12]. Moreover, UC-MSCs have been found to exert more pronounced immunosuppressive effects in mixed lymphocyte reactions compared to BM-MSCs or AT-MSCs [11,12].

The source of MSCs and the tissue microenvironment both have significant implications for immunomodulatory behavior [11]. While most MSCs exert immunosuppressive effects, recent findings suggest that under certain conditions they can also display immunostimulatory functions [12,13]. This duality is influenced both by intrinsic properties and external stimuli such as inflammatory cytokines. MSCs primed with IFN-γ or TNF-α often shift towards a more immunosuppressive phenotype, but this responsiveness may differ among tissue-derived MSCs [11,12]. UC-MSCs and placental MSCs may be more responsive to these priming signals, enhancing their therapeutic potential in inflammatory conditions [11]. Conversely, some MSC populations, such as those derived from the synovial membrane, may display a more limited range of immunomodulatory behavior or even promote immune activation under certain conditions [11].

In a similar manner to their parental cells, mesenchymal stem cell-derived extracellular vesicles (MSC-EVs) can modulate immune response, displaying both immunosuppressive and immunostimulatory properties [14]. MSC-EVs carry a complex cargo of MSC-sourced proteins, lipids, mRNAs, and microRNAs. MSC-EVs can inhibit T cell proliferation, promote expansion of immunosuppressive T regulatory cells (Tregs), suppress B cell activation, and modulate the activity of dendritic cells (DCs) and natural killer (NK) cells [14]. However, depending on the context, particularly the inflammatory milieu and the activation status of their parental MSCs, MSC-EVs can also enhance immune responses by stimulating maturation of antigen-presenting cells or by promoting release of inflammatory cytokines from NK cells, macrophages, neutrophils, DCs, and Th1 or Th17 lymphocytes [14].

The ability of MSCs and MSC-EVs to either suppress or stimulate immune response makes them potent therapeutic tools, capable of precisely modulating progression of inflammatory, autoimmune, and malignant diseases [12,13,14]. Accordingly, in recent years, MSCs and their EVs have gained significant attention as adjunctive or combinatorial components in CAR-based immunotherapy, owing to their potential to enhance therapeutic efficacy and minimize treatment-related adverse effects [15,16,17]. MSCs possess inherent tumor-homing capabilities and can modulate the phenotype and function of tumor-infiltrating leukocytes [18,19]. Through secretion of immunoregulatory cytokines and chemokines, MSCs can also influence the viability, activity, trafficking, phenotype, and effector functions of CAR-modified immune cells [16,19]. MSCs have potential applications as cellular carriers for immunomodulatory payloads, as supportive elements to enhance the trafficking and survival of CAR-modified leukocytes, or as tools to condition the tumor microenvironment toward a more pro-inflammatory state that promotes immune activation [16]. Although MSC-based approaches are still in the early stages of development, they offer promising strategies to overcome current limitations of CAR-based therapies, particularly in the context of solid tumors [18,19].

Accordingly, in this review, we highlight the current state of research on the effects of MSCs on CAR-expressing immune cells. We discuss the mechanisms by which MSCs might modulate anti-tumor immune response, we summarize findings obtained in studies which explored synergy between MSCs and CAR-modified leukocytes, and we identify key challenges and future directions in this rapidly evolving field. An extensive literature review was carried out in April 2025 across several databases (MEDLINE, EMBASE, and Google Scholar), covering research from 1990 to the present. Keywords used in the selection were as follows: “mesenchymal stem cells”, “mesenchymal stem cell-derived extracellular vesicles”, “Chimeric Antigen Receptor-based immunotherapy”, “CAR-expressing immune cells”, “CAR-based therapy of malignant diseases”, “signaling pathways”. All journals were considered, and an initial search retrieved 129 articles. The abstracts of all these articles were subsequently reviewed by two of the authors (VV and CRH) independently to check their relevance to the subject of this manuscript. Eligible studies had to delineate the molecular and cellular mechanisms that are involved in MSC-dependent modulation of CAR-expressing immune cells. Their findings are analyzed in this review.

## 2. Development, Phenotype, and Function of CAR-Expressing T Lymphocytes

CAR T cells typically comprise a heterogeneous population of CD4^+^ helper T cells (Th) and CD8^+^ cytotoxic T lymphocytes (CTLs), although enrichment for specific subsets can be performed to enhance therapeutic efficacy [2,3]. Following genetic modification, CAR constructs are introduced into T cells that have been previously isolated from the patient’s peripheral blood [4].

The CAR molecule consists of several modular domains, each with a distinct structural and functional role [20,21]. It is designed to emulate certain features of natural immune receptors while enhancing tumor specificity and cytotoxic activity [20,21]. The extracellular portion of the CAR serves as the antigen-recognition domain and typically comprises a single-chain variable fragment (scFv) derived from the variable regions of a monoclonal antibody [20]. This scFv domain is responsible for recognizing and binding to specific tumor-associated antigens, such as CD19 on malignant B cells, HER2 on breast cancer cells, or BCMA on malignant plasma cells [20]. The scFv includes both the heavy and light chains, connected via a flexible peptide linker that ensures proper folding and antigen-binding specificity [21]. In contrast to native T-cell receptors (TCRs), which require antigen presentation via major histocompatibility complex (MHC) molecules, CARs facilitate direct antigen recognition on the surface of tumor cells [21]. This MHC-independent mechanism makes CAR T cells particularly advantageous in targeting tumors that evade immune surveillance by down-regulating MHC expression [21]. Positioned beneath the scFv, the hinge or spacer region provides structural flexibility and optimal spatial orientation, enhancing the CAR’s ability to engage with target antigens effectively [21]. The transmembrane domain anchors the CAR in the T-cell membrane and contributes to receptor stabilization and signal transduction [21]. The intracellular signaling domain is the functional core of the CAR, initiating T-cell activation. This domain invariably contains the CD3ζ chain, which harbors immunoreceptor tyrosine-based activation motifs (ITAMs) essential for signal propagation upon antigen binding [21].

**Figure 1:**
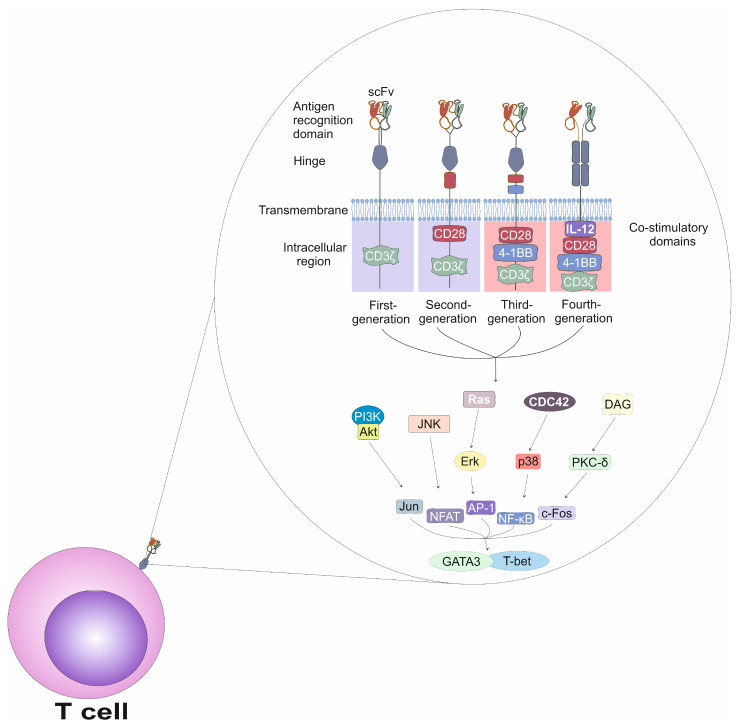
Structure of CAR receptors and the downstream intracellular signaling pathways triggered upon their activation in CAR T cells. Each CAR in CAR T lymphocytes is composed of distinct regions that together allow T cells to recognize and attack tumor cells more effectively. At the forefront of the CAR structure is the antigen-recognition domain, typically a single-chain variable fragment (scFv) derived from a monoclonal antibody. The scFv consists of heavy and light antibody chains which are joined by a flexible linker to preserve structure and antigen-binding capability. Located directly beneath the scFv is the hinge or spacer region, which grants the receptor flexibility and spatial reach, optimizing engagement between the CAR T cell and the tumor cell. The transmembrane domain acts as an anchor, embedding the CAR into the T-cell membrane. At the core of the CAR’s functionality lies the intracellular signaling domain, which drives T-cell activation upon antigen engagement. Central to this is the CD3ζ chain, which contains immunoreceptor tyrosine-based activation motifs (ITAMs) necessary for initiating intracellular signaling cascades. To date, researchers have developed four generations of CAR receptors in the generation of CAR T lymphocytes. The first-generation CARs included only the basic components (scFv, hinge, transmembrane, and CD3ζ) but lacked co-stimulatory domains. To address this shortcoming, second-generation CARs were developed by integrating one co-stimulatory domain, such as CD28 or 4-1BB (CD137), in addition to the CD3ζ chain. The third-generation CARs incorporated two co-stimulatory domains, commonly CD28 and 4-1BB, alongside CD3ζ. The fourth and most advanced generation of CARs, known as TRUCKs (T cells Redirected for Universal Cytokine-mediated Killing), are engineered to express immune-stimulating molecules such as the pro-inflammatory cytokine IL-12. These engineered cytokines allow CAR T cells not only to kill cancer cells directly but also to modulate the tumor microenvironment. Engagement of CAR receptor initiates a complex intracellular signaling cascade which activates multiple pathways in CAR T cells. Phosphoinositide 3-kinase (PI3K) generates PIP3 and activates AKT, which promotes survival and regulates metabolism of activated CAR T cells. Concurrently, DAG, produced by PLC-γ1-mediated hydrolysis of PIP2, activates Protein Kinase C (PKC), particularly PKC-θ, which is essential for NF-κB activation. DAG also facilitates activation of the Ras–MAPK pathway, including Ras, ERK, and JNK, which phosphorylate and activate transcription factor AP-1, composed of c-Fos and Jun. CDC42, a Rho-family GTPase, modulates actin cytoskeletal rearrangement and contributes to p38 MAPK activation. These signaling modules collectively lead to the activation of key transcription factors including NFAT, NF-κB, and AP-1, which orchestrate gene expression programs governing T cell proliferation and differentiation. Lineage-specific transcription factors such as T-bet and GATA3 are subsequently induced, directing differentiation of Th1 and Th2, respectively.

CAR designs have progressed through four distinct generations, each incorporating structural refinements aimed at improving T-cell activation, persistence, and anti-tumor efficacy (Figure 1) [20,21]. First-generation CARs consisted of an scFv, a hinge, a transmembrane domain, and a single intracellular signaling domain containing the CD3ζ chain, without any co-stimulatory elements. As a result, T cells expressing first-generation CARs exhibited limited in vivo proliferation [20,21]. Second-generation CARs addressed this limitation by incorporating one co-stimulatory domain, either CD28 or 4-1BB (CD137). This domain is in conjunction with CD3ζ, significantly enhancing survival and expansion of CAR T cells [20,21]. Third-generation CARs integrate two co-stimulatory domains, commonly CD28 and 4-1BB, thereby amplifying T-cell activation and promoting more-durable immune responses [20]. Fourth-generation CARs, also known as T cells Redirected for Universal Cytokine-mediated Killing (TRUCKs), retained the components of previous generations but were further engineered to express additional immune-activating molecules such as the pro-inflammatory cytokine IL-12 [20,21]. T cells which express fourth-generation CARs are able to efficiently modulate the tumor microenvironment and overcome immunosuppressive mechanisms employed by tumor cells [20,21].

Phenotypically, effective CAR T cells resemble memory T cells which are characterized by CD62L^+^CD45RO^+^CD3^+^ expression profiles and exhibit superior proliferative capacity and persistence in vivo compared to effector T cells [21]. Long-lived memory CAR T cells are believed to play a critical role in sustained anti-tumor surveillance, which is essential for preventing relapse in hematologic malignancies [21].

Activation of CAR T cells is initiated when the CAR binds its cognate antigen on the surface of a tumor cell, triggering a cascade of intracellular signaling events which mimic the physiological activation of T cells [1,2,3]. Activation of T cells is a tightly regulated process that involves an interplay between kinases, adaptor proteins and transcription factors [22]. The TCR is a heterodimeric structure composed of α and β chains, which are responsible for antigen recognition [22]. However, the TCR alone lacks intrinsic signaling capacity and relies on the activity of the associated CD3 complex, consisting of the CD3γ, CD3δ, CD3ε, and CD3ζ chains, which transduces signals into the cell [22]. TCR-driven signaling begins with phosphorylation of ITAMs in the CD3ζ chains by the lymphocyte-specific protein tyrosine kinase (Lck), which is associated with the cytoplasmic tail of the CD4 or CD8 co-receptors [22]. Upon binding to the doubly phosphorylated ITAMs of CD3ζ, zeta-chain-associated protein kinase 70 (ZAP-70) becomes phosphorylated and activated by Lck [22]. Activated ZAP-70 subsequently phosphorylates several adaptor proteins, including linker for activation of T cells (LAT) and SH2 domain-containing leukocyte protein of 76 kDa (SLP-76), which act as scaffolds to recruit additional signaling molecules, assembling the LAT signalosome [22]. The LAT signalosome serves as a hub for several downstream signaling pathways [22]. One of the major pathways involves the activation of phospholipase C-γ1 (PLC-γ1), which is recruited to LAT and activated through interleukin-2–inducible T cell kinase (ITK)-dependent phosphorylation [22]. Activated PLC-γ1 hydrolyzes phosphatidylinositol 4,5-bisphosphate (PIP2) into two secondary messengers: inositol 1,4,5-trisphosphate (IP3), which induces calcium release from the endoplasmic reticulum; and diacylglycerol (DAG), which activates protein kinase C-θ (PKC-θ) and Ras guanine nucleotide-releasing protein (RasGRP) [22]. The increase in intracellular calcium leads to the activation of calcineurin, which dephosphorylates and activates the transcription factor Nuclear factor of activated T cells (NFAT), enabling its translocation into the nucleus [22]. DAG-mediated signaling also leads to the activation of the Ras–Mitogen-activated protein kinase (MAPK) pathway, resulting in the activation of nuclear factor kappa-light-chain-enhancer of activated B cells (NF-κB) and activator protein 1 (AP-1), transcription factors which regulate the expression of genes essential for T cell proliferation, survival, differentiation, and effector functions [22].

The result of CAR T cell activation is a robust response that includes expansion of CAR T cells, massive release of cytotoxic granules (perforin and granzymes) from CAR CD8+CTLs, and increased production of pro-inflammatory cytokines (IFN-γ, IL-2, TNF-α) from CAR CD4+ T cells [2,3]. These effector mechanisms enable CAR T cells to kill tumor cells directly and recruit other components of the immune system to enhance the anti-tumor response [2,3].

## 3. Molecular Mechanisms Responsible for MSC-Dependent Modulation of Tumor-Infiltrating T Cells

**Figure 2:**
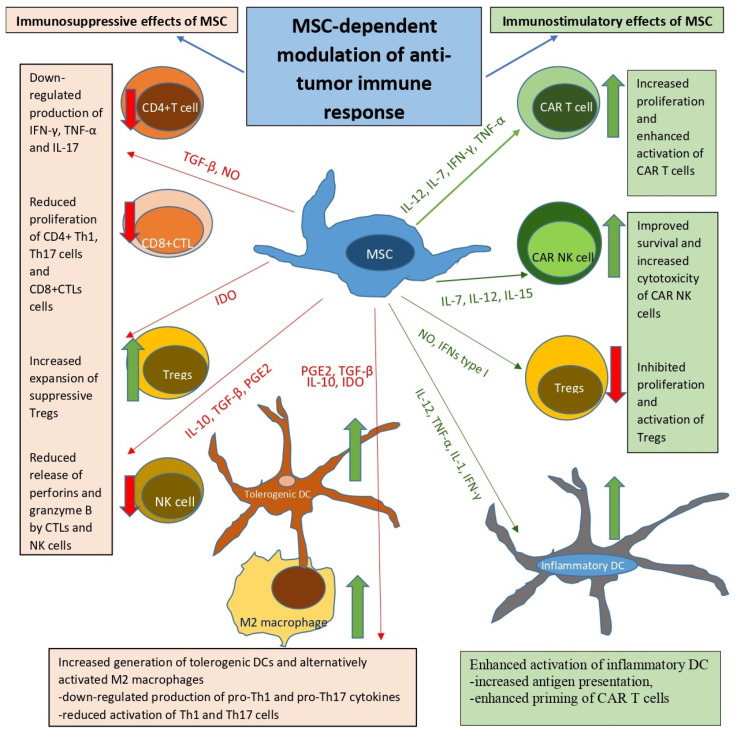
Pleiotropic immunomodulatory functions of MSCs in anti-tumor immunity. MSCs exhibit a dual role in modulating the anti-tumor immune response, with both immunosuppressive and immunostimulatory mechanisms being involved. MSC-derived immunosuppressive factors (IL-10, TGF-β, PGE2, IDO) reduce the proliferation, activation, and effector functions of CD4+Th1 and Th17 lymphocytes, down-regulate production of inflammatory cytokines (IFN-γ, TNF-α, IL-17), inhibit release of perforins and granzyme B by CTLs and NK cells, induce generation and expansion of Tregs, tolerogenic DCs and M2 alternatively activated macrophages, attenuating anti-tumor immune response. Conversely, MSCs may exert immunostimulatory effects under certain contexts. MSC-derived inflammatory cytokines (IL-1, IL-7, IL-12, IL-15, IFN-γ, TNF-α) may improve antigen-presenting properties of DCs, increase proliferation and activation of CAR T cells and CAR NK cells, while MSC-sourced NO and interferons type I may suppress expansion of Tregs, thereby potentiating anti-tumor immunity.

MSCs can exert either immunosuppressive or immunostimulatory effects on CD4^+^ and CD8^+^ T cells within the tumor microenvironment (Figure 2) [18]. MSC-mediated immunosuppression often involves the inhibition of T helper 1 (Th1) cell polarization, primarily through secretion of the soluble mediators prostaglandin E2 (PGE2), transforming growth factor-β (TGF-β) and indoleamine 2,3-dioxygenase (IDO) [18]. These factors down-regulate the expression of T-bet, a key transcription factor required for Th1 differentiation, and reduce the production of Th1-related inflammatory cytokine IFN-γ, thereby attenuating Th1 cell-driven immune responses [18]. Similarly, MSCs suppress the differentiation and function of Th17 cells by secreting IL-10 and TGF-β, and by depleting tryptophan via IDO activity [19]. This depletion destabilizes the expression of retinoic acid receptor-related orphan receptor gamma t (RORγt), the master transcription factor driving Th17 lineage commitment [19]. Through these mechanisms, MSCs contribute to the reduction of inflammation and the suppression of excessive Th1/Th17 cell-mediated cytotoxicity within tumors [18,19].

While MSCs are predominantly recognized for their immunosuppressive roles, emerging evidence indicates that, under certain conditions, they can reprogram the tumor microenvironment (TME) to favor pro-inflammatory, T cell-permissive responses (Table 1) [19]. Exposure to the pro-inflammatory cytokines IFN-γ, IL-12, and TNF-α has been shown to improve the antigen presenting properties of DCs and enhance the activation and cytolytic function of tumor-infiltrating lymphocytes (TILs) [19]. Elevated levels of tumor-associated neoantigens, along with efficient cross-presentation by DCs, can sustain robust T cell responses by providing continuous antigenic stimulation [19]. When exposed to inflammatory signals, MSCs may produce nitric oxide (NO), reactive oxygen species (ROS), and type I interferons, which impair the function of suppressive immune cells while enhancing local inflammation, thereby promoting T cell activation and effector function [23]. Additionally, a metabolically supportive microenvironment, characterized by adequate oxygenation, glucose availability, and low lactate concentrations, can preserve T cell viability and functionality while preventing exhaustion [23]. Moreover, the absence or therapeutic blockade of immune checkpoint pathways, such as programmed death-ligand 1 (PD-L1), cytotoxic T-lymphocyte–associated antigen 4 (CTLA-4), and other inhibitory receptors, is critical for sustaining T cell effector activity [18,19]. Together, these factors contribute to the establishment of an inflammatory milieu conducive to sustained and effective anti-tumor T cell responses [18].

Under appropriate inflammatory priming, MSCs may function as immune modulators that promote reprogramming of the TME to support anti-tumor immunity [19]. In this context, MSCs can act as immunological adjuvants by enhancing T cell infiltration, survival, and functionality within solid tumors [23]. MSCs facilitate the recruitment and retention of TILs through the secretion of chemokines such as CCL5 (RANTES), CXCL9, and CXCL10 [23]. These chemokines interact with CCR5 and CXCR3 receptors on activated T cells, directing their migration toward inflamed tumor tissues [23]. Additionally, MSCs engineered to overexpress T cell-attracting chemokines or pro-inflammatory mediators have demonstrated an improved capacity to enhance TIL accumulation in preclinical tumor models [24]. This chemotactic enhancement not only increases intra-tumoral CD8^+^ T cell density but also supports the formation of immune hotspots or tertiary lymphoid structures [24]. Beyond T cell recruitment, MSCs can support the survival, expansion, and cytolytic activity of TILs [24,25]. Furthermore, they may mitigate TIL exhaustion by modulating the expression of inhibitory receptors such as programmed death-1 (PD-1) and T-cell immunoglobulin and mucin-domain containing-3 (TIM-3) [23]. This functional restoration could help recover the anti-tumor potential of TILs that have become dysfunctional due to chronic antigen exposure within the TME [23].

## 4. Signaling Pathways Involved in the Cross-Talk Between MSCs, CAR T Cells, and Tumor Cells

Holthof and colleagues investigated molecular mechanisms involved in the cross-talk between BM-MSCs, CAR T cells, and multiple myeloma (MM) tumor cells [26]. For this purpose, a panel of 10 CAR T cells targeting BCMA, CD38, and CD138 with varying affinities were evaluated for their cytotoxic efficacy against MM cells in the presence of BM-MSCs [26]. Holthof and co-workers observed that BM-MSCs can shield MM cells from CAR T cells with lower affinity and moderate lytic activity [26]. High-affinity BCMA- and CD38-specific CAR T cells demonstrated robust cytotoxic activity against MM cells, effectively lysing target cells even in the presence of BM-MSCs, while CAR T cells with moderate or lower affinity to BCMA, CD38, and CD138 showed reduced lytic activity against MM cells [26] Among different BCMA-, CD38-, and CD138-specific CAR T cells, BM-MSCs did not show suppressive effects against BCMA^C11D5.3^-CAR T cells and BBz-CD38B1-CAR T cells, as evidenced by the unchanged or even enhanced secretion of the key effector, the tumorotoxic molecules IFN-γ and granzyme B, in both the UM9 MM cell line and in primary MM cells which were derived from MM patients [26]. The BCMA^C11D5.3^-CAR T cells were directed against BCMA protein that was highly expressed on malignant plasma cells. These CAR T cells utilize the C11D5.3 scFv for antigen recognition, which is linked to an intracellular signaling domain composed of CD3ζ and the co-stimulatory domain 4-1BB, enhancing T cell activation, persistence, and memory-like responses [26]. Similarly, BBz-CD38B1-CAR T cells target CD38, a transmembrane glycoprotein abundantly expressed on MM cells [26]. The CD38B1 scFv confers antigen specificity, while the CAR construct includes the 4-1BB co-stimulatory domain and the CD3ζ signaling domain, promoting potent cytotoxic responses upon antigen engagement [26]. Both of these CAR T cells were capable of producing IFN-γ and granzyme B in the presence of BM-MSCs, leading to the targeted lysis of MM cells [26]. These findings indicate that although BM-MSCs do not uniformly suppress CAR T cell activity, they can exert a partial and selective inhibitory effect depending on the CAR construct and target antigen involved [26].

BM-MSCs provided protection to CAR T-treated MM cells through a cell–cell contact-dependent manner [26]. This protection was associated with up-regulation of the anti-apoptotic proteins Survivin and myeloid cell leukemia 1 (Mcl-1) in MM cells, which impaired CAR T cell-induced cytotoxicity by suppressing caspase 3 and caspase 7-driven apoptosis and inhibiting sequestration of Bim and Noxa proteins, thereby preventing mitochondrial outer membrane permeabilization and subsequent activation of the intrinsic apoptotic cascade [27]. Importantly, Holthof and colleagues revealed that BM-MSC-mediated resistance could be effectively counteracted by FL118, a small-molecule inhibitor targeting multiple anti-apoptotic proteins in MM cells [26,28]. FL118 inhibits Survivin and Mcl-1 by selectively suppressing their gene transcription and promoting proteasome-mediated degradation, thereby disrupting their ability to inhibit caspase activation and neutralize pro-apoptotic proteins, ultimately restoring the apoptotic sensitivity of MM cells to CAR T cell-mediated killing [26,28]. These findings indicated that overcoming BM-MSC-induced immune resistance may be achievable through the inhibition of these key anti-apoptotic molecules in MM cells.

Zanetti and colleagues investigated the interaction between BM-MSCs and CD19-specific CAR T lymphocytes (CD19-CAR T cells) [31]. They isolated BM-MSC from five pediatric B-ALL patients (BM-MSCs^B-ALL^), including individuals with ETV6/RUNX1 and MLL-rearranged subtypes, and compared it with age-matched healthy donor (HD) BM-MSC^HD^ [31]. Both BM-MSCs^B-ALL^ and BM-MSC^HD^ exhibited similar morphology, immunophenotype, differentiation potential, immunosuppressive and anti-inflammatory properties, but BM-MSCs^B-ALL^ displayed reduced proliferative capacity compared to BM-MSC^HD^ [31]. Importantly, BM-MSCs^B-ALL^ and BM-MSCs^HD^ did not impair the cytotoxic activity or cytokine production of CD19-CAR T cells in vitro [31]. When co-cultured with B-ALL cell lines and primary B-ALL cells, CD19-CAR T cells maintained their ability to lyse target cells and produce pro-inflammatory cytokines, regardless of the presence of BM-MSCs from either HD or B-ALL patients [31]. Furthermore, in a preclinical B-ALL xenograft model, CD19-CAR T-cells effectively controlled the growth of human pre-B ALL cells (NALM6 cells), irrespective of the presence of BM-MSCs^B-ALL^ and BM-MSCs^HD^ [31]. These findings suggest that, while BM-MSCs^B-ALL^ retain their immunosuppressive effects on conventional T-cells, they do not compromise the activity of CD19-CAR T cells, suggesting that CD19-CAR T cells can effectively target and eliminate B-ALL cells even in the presence of the immunosuppressive bone marrow microenvironment [31].

## 5. MSC-Based Enhancement of CAR T-Cell-Based Therapy for Solid Tumors

McKenna and co-workers explored a novel approach to enhance the efficacy of CAR T-cell-based therapy for solid tumors by leveraging MSCs as delivery vehicles for oncolytic immunotherapy [26]. The researchers utilized a combinatorial adenoviral vector (CAd) comprising an oncolytic adenovirus (OAd) and a helper-dependent adenovirus (HDAd) engineered to express IL-12 and a PD-L1 blocking antibody [26]. The combination therapy demonstrated significant efficacy in both in vitro and in vivo models [26]. In 3D tumor spheroid assays, the co-administration of CAd-infected MSCs and CAR T cells led to substantial tumor cell death [26]. In orthotopic lung cancer mouse models, this combinatorial treatment suppressed tumor growth more effectively than CAR T-cell therapy alone [26]. Additionally, the combination of MSCs and CAR T-cell-based therapy increased the overall numbers of human CAR T cells in experimental animals and enhanced their capacity for secretion of inflammatory and tumorotoxic cytokines, indicating that CAd-infected MSCs managed to improve activation and persistence of CAR T cells in the tumor microenvironment [26]. Mechanistically, CAd-infected MSCs facilitated the spread of the oncolytic virus within the tumor microenvironment, leading to the release of IL-12 and the PD-L1 blocker [26]. IL-12 is a potent immunostimulatory cytokine that promotes CAR T-cell activation, while the PD-L1 blocker enhances CAR T-cell responses by inhibiting the PD-1/PD-L1 checkpoint pathway [27]. The combination of IL-12 and PD-L1 blocker resulted in increased T-cell infiltration into tumors and enhanced their effector functions, thereby improving the overall anti-tumor response [26]. Results obtained in this study demonstrated the potential of MSCs as effective delivery vehicles for oncolytic immunotherapy, capable of overcoming the challenges associated with delivering therapeutic agents to solid tumors [26]. By combining the tumor-targeting capabilities of MSCs with the oncolytic and immunomodulatory properties of the CAd, this approach offers a promising strategy to enhance the efficacy of CAR T-cell therapies for solid tumors [26].

In line with these findings are the results recently obtained by Hombach and colleagues, who investigated the potential of genetically engineered, IL-7- and IL-12-over-expressing MSCs to enhance the efficacy of CAR T cells in treating colorectal cancer [28]. Hombach and co-workers demonstrated that co-culturing anti-carcinoembryonic antigen (CEA)-specific CAR-expressing T cells with CEA-positive LS174T colorectal cancer cells in the presence of IL-7/IL-12-engineered MSCs produced significantly higher amounts of pro-Th1 inflammatory cytokines IFN-γ and TNF-α [28]. This cytokine profile indicated a shift towards a Th1 cell-driven immune response, which is associated with effective elimination of malignant cells [28]. Furthermore, the engineered MSCs promoted the release of IL-2, which is known to support activation and survival of Th1 cells [28,31]. This mutual activation of MSCs and CAR T cells led to increased CAR T cell proliferation, reduced activation-induced cell death, and enhanced cytotoxic activity against tumor cells [28]. In vivo experiments using a xenograft mouse model of colorectal cancer further validated these findings [28]. Mice treated with IL-7/IL-12-engineered MSCs in combination with CAR T cells exhibited significantly reduced tumor growth [28]. These findings suggest that genetically modified MSCs capable of delivering IL-7 and IL-12 to the tumor site can effectively modulate the tumor microenvironment by promoting Th1 cell-driven immune response that supports CAR T cell activity [28]. This strategy represents a promising avenue for improving the outcomes of CAR T-cell therapies in solid tumors, where challenges such as immunosuppressive microenvironments often limit therapeutic efficacy.

McKenna and colleagues investigated an innovative strategy to enhance the efficacy of CAR T-cell-based therapies for solid tumors by employing MSCs as delivery vehicles for oncolytic immunotherapy [32]. These researchers utilized a combinatorial adenoviral vector (CAd), consisting of an oncolytic adenovirus (OAd) and a helper-dependent adenovirus (HDAd), engineered to co-express interleukin-12 (IL-12) and a programmed death-ligand 1 (PD-L1) blocking antibody [32]. This therapy demonstrated substantial efficacy in both in vitro and in vivo models. In three-dimensional (3D) tumor spheroid assays, co-administration of CAd-infected MSCs and CAR T cells resulted in marked tumor cell death [32]. In orthotopic lung cancer mouse models, the combinatorial treatment significantly suppressed tumor growth compared to CAR T-cell therapy alone [32]. Additionally, the combination therapy led to an increased number of human CAR T cells within experimental animals and enhanced their secretion of pro-inflammatory and cytotoxic cytokines, indicating that CAd-infected MSCs improved both the activation and persistence of CAR T cells in the TME [32].

Mechanistically, CAd-infected MSCs facilitated the intra-tumoral spread of the oncolytic virus, enabling the localized release of IL-12 and the PD-L1 blocking antibody [32]. IL-12 is a potent immunostimulatory cytokine that enhances CAR T cell activation and expansion, while blockade of PD-L1 augments T cell responses by inhibiting the PD-1/PD-L1 immune checkpoint pathway [33]. The combined expression of IL-12 and the PD-L1 inhibitor led to increased T-cell infiltration into the tumor and enhanced their effector functions, collectively contributing to an improved anti-tumor response [32]. These findings underscore the potential of MSCs as effective delivery platforms for oncolytic immunotherapy, capable of addressing the challenges associated with therapeutic delivery in solid tumors [32]. By integrating the tumor-homing properties of MSCs with the immunostimulatory and oncolytic functions of the CAd system, this approach represents a promising strategy to potentiate CAR T-cell therapy for solid malignancies [32].

Supporting these findings, Hombach and colleagues recently evaluated the use of genetically engineered IL-7- and IL-12-overexpressing MSCs to augment the efficacy of CAR T cells in colorectal cancer [29]. Co-culturing of anti-carcinoembryonic antigen (CEA)-specific CAR T cells and CEA-positive LS174T colorectal cancer cells in the presence of IL-7/IL-12-engineered MSCs resulted in enhanced CAR T-cell-driven anti-tumor immune response. Upon their co-culture with IL-7- and IL-12-overexpressing MSCs, CAR T cells significantly increased secretion of tumorotoxic, pro-Th1 cytokines (IFN-γ and TNF-α) [29]. Moreover, IL-7- and IL-12-overexpressing MSCs promoted the release of IL-2 in CAR T cells [29]. IL-2 is a cytokine which supports the activation, survival, and expansion of Th1 cells [30]. Accordingly, the interaction between IL-7/IL-12-overexpressing MSCs and CAR T cells improved proliferation of CAR T lymphocytes, reduced activation-induced cell death, and increased their cytotoxicity against tumor cells [29].

In vivo experiments using a xenograft mouse model of colorectal cancer further validated these findings [29]. Mice treated with IL-7/IL-12-engineered MSCs in combination with CAR T cells exhibited significantly reduced tumor growth [29]. These findings suggest that genetically modified MSCs capable of delivering IL-7 and IL-12 to the tumor site can effectively modulate the TME, by promoting Th1 cell-driven immune response that supports CAR T-cell activity [29]. This strategy represents a promising avenue for improving the outcomes of CAR T-cell therapies in solid tumors, where challenges such as immunosuppressive microenvironments often limit therapeutic efficacy.

## 6. Phenotype and Functional Properties of CAR NK Cells

CAR NK cells are genetically modified NK cells endowed with a synthetic receptor that enables them to specifically recognize and eliminate tumor cells expressing a chosen antigen [34]. They can be derived from various sources, including peripheral blood, umbilical cord blood (UCB), induced pluripotent stem cells (iPSCs), or the NK-92 cell line [34,35]. Phenotypically, CAR NK cells maintain the characteristic markers of NK cells such as high expression of CD56, CD16, and NKG2D receptors, and lack of the CD3 molecule, distinguishing them from CD3^+^T lymphocytes [34]. CARs engineered into NK cells are structurally similar to those used in CAR T cells but with important modifications to better suit NK cell biology [34]. Like in CAR T cells, the CAR receptor in CAR NK cells is composed of several modular domains: an extracellular antigen-recognition domain, a hinge or spacer region, a transmembrane domain, and one or more intracellular signaling domains [34]. The extracellular domain of a CAR in NK cells typically features scFv derived from a monoclonal antibody, which is responsible for direct recognition of tumor-associated antigens in an MHC-independent manner [34]. This feature is especially important for NK cell-dependent elimination of malignant cells that down-regulate MHC class I proteins in order to evade CTLs-mediated immune response [35]. Beneath the scFv lies the hinge or spacer region, which provides flexibility and proper spatial orientation for antigen binding [34,35]. The transmembrane domain of CAR receptors in NK cells is commonly sourced from NK cell-specific proteins NKG2D or CD16, while the intracellular signaling domain contains NK cell activating receptors DAP10, DAP12, or 2B4 (CD244) [34,35]. These molecules naturally mediate activating signals in NK cells through their intracellular ITAMs [34]. Incorporating these NK-compatible signaling domains enhances the cytotoxic function, proliferation, and survival of CAR NK cells without compromising their safety profile [34,35]. CAR NK cells combine the antigen specificity of synthetic CARs with the innate cytotoxicity and safety profile of NK cells [34]. They exert CAR-mediated cytotoxicity, where engagement of the scFv with its target tumor antigen triggers intracellular signaling that leads to the release of cytotoxic granules (perforins and granzymes) and pro-inflammatory cytokines (IFN-γ and TNF-α) [34,35]. Additionally, CAR NK cells retain their natural cytotoxicity, allowing them to respond to stressed or malignant cells through NKG2D, NKp30, and DNAM-1 receptors [34]. This dual killing capability enhances their effectiveness against tumor heterogeneity and antigen-loss variants of malignant cells, making CAR NK therapy a highly promising avenue in cancer immunotherapy for hematologic malignancies and solid tumors [34,35].

## 7. MSC-Dependent Modulation of CAR-Expressing NK Cells

Upon engraftment into the tumor microenvironment, MSCs are able to modulate the cytotoxicity, proliferation, and cytokine secretion of NK cells in a contact-dependent and paracrine manner [23]. Soluble mediators such as PGE2, TGF-β, and IDO play pivotal roles in down-regulating activating NK receptors like NKG2D and NKp30, thus impairing target cell recognition [18]. Additionally, MSC-EVs can deliver miRNAs and immunomodulatory proteins that influence NK cell function [24]. These interactions collectively result in reduced secretion of granzyme B and IFN-γ by NK cells, impairing their cytolytic function against tumor cells [23].

However, depending on the inflammatory milieu and priming signals, MSCs may also enhance NK cell recruitment and survival, indicating a context-dependent regulatory role [19,23]. MSC-derived chemokines CXCL9, CXCL10, and CCL5 interact with CXCR3 and CCR5 on NK cells to promote their directed migration into tumor tissues [19]. This chemokine-driven infiltration ensures that NK cells are positioned close to malignant cells, increasing the likelihood of cytotoxic engagement [19]. When primed by inflammatory cytokines (IFN-γ, TNF-α, IL-1β), MSCs secrete IL-15, IL-12, and type I interferons, which are critical for maintaining NK cell cytotoxicity and survival in hostile tumor settings [19,23]. MSC-derived IL-15 plays a central role in sustaining NK cell survival and proliferation, particularly in nutrient-deprived or immunosuppressive tumor environments [19]. Importantly, MSC-sourced IL-15 not only enhances viability and expansion of NK cells, but also up-regulates expression of cytotoxic molecules (perforin, granzyme B, and Fas ligand (FasL)), thereby augmenting their ability to directly kill tumor cells [23]. Furthermore, MSC-derived interferon-beta (IFN-β), miR-155, and miR-21 can enhance the expression of activating receptors (NKG2D, NKp30, and DNAM-1) on NK cells [19]. MSC-sourced CXCL10 (IP-10) facilitates NK cell recruitment into the tumor microenvironment and supports NKG2D expression through CXCR3-mediated activation [19,23]. Together, these MSC-derived factors reprogram tumor-infiltrated NK cells toward a more activated, cytotoxic phenotype, improving their ability to recognize and eliminate tumor cells [19,23].

Holthof and colleagues analyzed the molecular mechanisms by which BM-MSCs protected MM cells from CAR NK cell-mediated cytotoxicity [36]. BM-MSCs exerted their protective influence by up-regulating anti-apoptotic proteins Survivin and Mcl-1 in MM cells [26]. These molecules inhibited NK cell-dependent activation of apoptotic pathways in MM cells [27]. To counter BM-MSC-induced immune resistance, the researchers tested two strategies. NK cells were genetically modified to express either a CD38-specific chimeric antigen receptor (CD38-CAR) or a novel TRAIL variant (TRAILv) engineered to selectively bind and activate the death receptor DR5 [36]. Both modified NK cell populations demonstrated significantly improved cytotoxicity against MM cells, even in the presence of immunosuppressive BM-MSCs [36]. Notably, CD38-CAR NK cells were found to massively release cytotoxic granules and inflammatory, pro-Th1 cytokines, while TRAILv-expressing NK cells more effectively activated DR5-dependent apoptosis of MM cells [36]. Furthermore, in a similar manner to that which they observed during the evaluation of BM-MSC–CAR T cell cross-talk [26], Holthof and colleagues demonstrated that use of the FL118 inhibitor, which suppressed activity of Survivin and Mcl-1, successfully overcame BM-MSC-mediated resistance of MM cells to CAR NK cell-induced apoptosis [36]. These findings suggest that a combination of CD38-CAR or TRAILv-expressing NK cells with FL118-based treatment of MM cells could efficiently reverse BM-MSCs-dependent immunosuppression, and should therefore be further explored as a potentially novel approach to enhance CAR NK cell-based therapy for malignant diseases [36].

## 8. Tumor-Suppressive Effects of CAR-Expressing MSCs

Although MSCs are usually recognized for their immunomodulatory properties, accumulating evidence also supports their intrinsic tumor-suppressive capabilities, depending on tumor type, MSC origin, and the microenvironmental context [13,19,23]. MSCs can exert anti-tumor effects through multiple mechanisms, including secretion of antiproliferative factors, induction of tumor cell apoptosis, inhibition of angiogenesis, and modulation of immune responses toward tumor rejection [13,19,23]. MSCs may up-regulate the anti-angiogenic molecules thrombospondin-1 (TSP-1) and endostatin, impairing neovascularization within the tumor microenvironment [37,38]. The resultant disruption of nutrient and oxygen supply contributes to the suppression of tumor progression [37,38]. Additionally, MSCs secrete various bioactive molecules that directly inhibit proliferation and induce apoptosis of tumor cells [23]. MSC-derived tumor necrosis factor-related apoptosis-inducing ligand (TRAIL), Dickkopf-1 (DKK1), and IL-24 trigger caspase-dependent apoptotic pathways in cancer cells [13,14]. Additionally, MSCs can induce cell cycle arrest in tumor cells through the release of IFN-β and cell cycle inhibitors, while MSC-derived miR-155 and miR-21 can suppress proliferation of malignant cells through the inhibition of phosphoinositide 3-kinase (PI3K)/Akt and Wnt/β-catenin signaling pathways [23].

Aliperta and colleagues described a novel approach to immunotherapy for acute myeloid leukemia (AML) in which genetically modified human MSCs were utilized as autonomous factories for the production of anti-CD33-anti-CD3 bi-specific antibodies (MSCs^anti-CD33-anti-CD3^) [39]. These bi-specific antibodies were engineered to redirect CD3^+^T cells against CD33-expressing leukemic cells, offering a continuous and localized delivery mechanism that overcame the limitations of traditional bi-specific antibodies, which require continuous infusion due to their short half-lives [39]. MSCs^anti-CD33-anti-CD3^ were capable of efficiently redirecting human T cells against AML cell lines expressing varying levels of CD33 [39]. In vitro assays demonstrated that T cells, when exposed to MSCs^anti-CD33-anti-CD3^, effectively lysed CD33^+^ AML cells, indicating the immunostimulatory properties of MSCs^anti-CD33-anti-CD3^ [39]. In order to enhance T cell-driven anti-tumor immune response, the engineered MSCs^anti-CD33-anti-CD3^ were further modified to express the co-stimulatory molecule 4-1BBL [39]. This modification led to increased secretion of pro-inflammatory cytokines TNF-α and IFN-γ, and promoted greater T-cell expansion, thereby augmenting the anti-tumor efficacy of the redirected T cells [39]. The presence of 4-1BBL on MSCs provided a co-stimulatory signal that enhanced the activation and proliferation of T cells, even when the target AML cells expressed low levels of CD33 [39]. Importantly, the therapeutic potential of this approach was confirmed in vivo, in a mouse model of AML [39]. Mice co-injected with AML cells, T cells, and 4-1BBL-expressing MSCs^anti-CD33-anti-CD3^ exhibited significant protection against leukemia, with no signs of disease progression [39]. This outcome underscores the efficacy of MSCs^anti-CD33-anti-CD3^ as a vehicle for localized and sustained production and delivery of anti-CD33-anti-CD3 bi-specific antibodies, providing a promising strategy for targeted T cell-based immunotherapy for AML [39].

**Figure 3:**
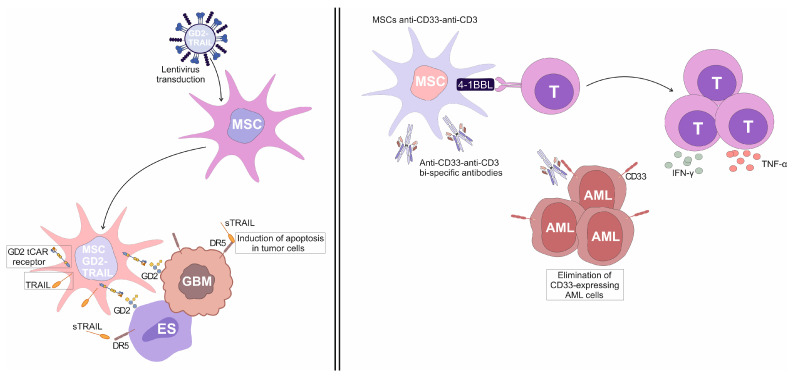
The use of genetically engineered MSCs in CAR T-cell-based immunotherapy for malignant diseases. GD2 and TRAIL-expressing MSCs (MSCs^GD2-TRAIL^) were generated when MSCs were transduced with lentiviral vectors encoding a truncated form of the anti-GD2 chimeric antigen receptor (GD2 tCAR) and the pro-apoptotic TRAIL molecule. MSCs^GD2-TRAIL^ were used for target elimination of GD2-positive glioblastoma (GBM) and Ewing’s sarcoma (ES) cells. TRAIL was both expressed on the membrane of MSCs^GD2-TRAIL^ and secreted from these cells as soluble TRAIL (sTRAIL). GD2 tCAR was used for selective targeting of GD2-expressing GBM and ES cells, while TRAIL bound to the DR5 receptor on GD2-expressing GBM and ES tumor cells, inducing their apoptosis. Similarly, genetically engineered human MSCs designed to produce anti-CD33-anti-CD3 bi-specific antibodies and express the co-stimulatory molecule 4-1BBL (MSCs^anti-CD33-anti-CD3^) markedly improved the CD3^+^ T cell–mediated killing of CD33-positive acute myeloid leukemia (AML) cells. The expression of 4-1BBL on these MSCs provided essential co-stimulatory signals that enhanced T cell activation and proliferation. T cells primed with MSCs^anti-CD33-anti-CD3^ showed rapid expansion and increased production of pro-inflammatory cytokines TNF-α and IFN-γ, resulting in effective elimination of CD33-expressing AML cells.

Golinelli and colleagues used genetically engineered GD2- and TRAIL-expressing MSCs (MSCs^GD2-TRAIL^) for target elimination of GD2-positive glioblastoma (GBM) cells [40]. They successfully transduced MSCs with lentiviral vectors encoding a truncated form of the anti-GD2 chimeric antigen receptor (GD2 tCAR) and pro-apoptotic TRAIL molecule, obtaining MSCs^GD2-TRAIL^ (Figure 3). The GD2 tCAR lacked intracellular signaling domains but retained the extracellular GD2-binding domain, enabling selective targeting of GD2-expressing GBM cells [40]. In vitro analyses demonstrated that MSCs^GD2-TRAIL^ effectively delivered TRAIL to GBM cells, initiating apoptosis. MSCs^GD2-TRAIL^ were also able to secrete a significant amount of soluble TRAIL (sTRAIL), indicating their potential to eliminate GD2-expressing tumor cells in a paracrine manner [40].

In addition to its effect on GBM cells, the GD2 and TRAIL bi-functional strategy also enhanced the specificity and efficacy of MSC-based therapies against GD2-expressing Ewing’s sarcoma (ES) cell lines (TC71, A673, and RD-ES) [41]. The TC71 cell line, which exhibited the highest GD2 expression, showed the strongest interaction with MSCs^GD2-TRAIL^ [41]. This binding was confirmed through cell-to-cell interaction assays, where MSCs^GD2-TRAIL^ formed aggregates with ES cells, indicating specific targeting mediated by GD2 tCAR [41]. Furthermore, MSCs^GD2-TRAIL^ released sTRAIL that induced apoptosis in GD2-expressing ES cells more efficiently than recombinant human TRAIL (rhTRAIL), underscoring the advantages of using MSCs^GD2-TRAIL^ for sustained delivery of sTRAIL in a tumor microenvironment of GBM, ES, and other GD2-expressing malignancies [41].

## 9. MSC-Mediated Modulation of CAR-Engineered Immune Cells Within the Tumor Microenvironment: Current Challenges and Future Perspectives

MSCs are generally well-tolerated, but concerns about their potential to support tumor progression still exist and have to be addressed [13,23,24,42]. To ensure the safety of MSC-based therapy in patients suffering from malignant diseases, this risk must be carefully mitigated through a combination of preclinical modeling, cell engineering, and treatment design [13,19,23,24]. One of the primary strategies to reduce the tumor-promoting risk of MSCs involves genetic or pharmacologic modification of MSCs to limit their ability to support tumor growth [24,42]. MSCs can be engineered to down-regulate synthesis of immunosuppressive cytokines and to overexpress tumor-suppressive molecules, such as interferons, pro-inflammatory cytokines and pro-apoptotic factors, enabling their delivery to the tumor microenvironment [42]. Additionally, incorporating “suicide genes” like inducible caspase-9 allows for the selective elimination of MSCs after they have delivered their therapeutic payload, preventing prolonged interaction with the tumor microenvironment [24,42].

Irradiation of MSCs prior to their administration offers a strategic approach to preserve their therapeutic efficacy while minimizing the risk of long-term immunosuppression that could hinder anti-tumor immune responses [24,43]. When exposed to a controlled dose of ionizing radiation (usually in a range of between 15 and 20 Grays), MSCs lose their ability to proliferate, effectively preventing their long-term engraftment, stromal integration, or potential transformation into tumor-supportive cell types [43]. However, and crucially, irradiation does not immediately compromise the cells’ secretory activity, allowing them to retain their short-term therapeutic functions [24,43]. These include the release of anti-inflammatory and regenerative factors which are essential for transient immune modulation, tissue repair, and support of hematopoietic recovery in clinical settings, such as for treatment of graft-versus-host disease or chemotherapy-induced injury [24,43]. By limiting the duration of MSC viability, irradiation also constrains the immunosuppressive influence of MSCs on CD8^+^ CTLs and NK cells [43,44]. Furthermore, irradiated MSCs undergo stress responses that result in the up-regulation of damage-associated molecular patterns (DAMPs) which act as immunostimulatory surface molecules, activating innate immune cells within the TME [44]. This dual effect of maintaining therapeutic benefit while avoiding persistent immune dampening makes irradiated MSCs especially valuable in oncological applications, where preserving host immune surveillance against malignancies is a priority [24,43,44].

Alternatively, researchers might utilize MSC-EVs rather than the cells themselves [45]. MSC-EVs can carry therapeutic cargo such as miRNAs or drugs without the risk of direct cellular interactions that might favor tumor growth [45]. Importantly, the design of clinical protocols should incorporate close monitoring and risk management, ensuring that the therapeutic benefits of MSCs and their EVs can be harnessed without compromising patient safety [45].

## 10. Conclusions

MSC-based modulation of CAR-engineered immune cells offers a promising strategy to enhance the efficacy, specificity, and safety of immunotherapy for malignant diseases [16]. By leveraging the immunomodulatory properties and tumor-homing capabilities of MSCs, this approach has the potential to improve trafficking of CAR-expressing T lymphocytes and NK cells to tumor sites, to modulate the tumor microenvironment and to reduce off-target toxicity [16,26,29,36]. Furthermore, engineered MSCs can serve as localized delivery vehicles for immunoregulatory agents that support function and persistence in CAR-engineered immune cells [16,40,41]. However, the immunosuppressive nature of MSCs and their potential to support tumor progression necessitates rigorous safety measures [42]. Strategies such as genetic engineering to limit immunosuppressive factors, incorporation of suicide genes, and pre-administration irradiation can mitigate these risks while preserving therapeutic benefit [24,43]. The use of MSC-EVs also offers a cell-free alternative with reduced safety concerns [45]. As this field advances, carefully designed preclinical models and clinical trials will be critical to optimize the cross-talk between MSCs and CAR-engineered immune cells, ensuring durable, safe, and effective cancer immunotherapy.

## Figures and Tables

**Table 1:** MSC-based modulation of CAR T-cell-driven anti-tumor immune response.

MSC-Based Immunomodulation	Molecules Responsible for MSC-Dependent Effects	Impact on CAR T Cells	Refs
Inhibition of Th1 polarization	PGE2, TGF-β, IDO	Down-regulated expression of T-bet, reduced production of IFN-γ, reduced cytotoxicity	[18]
Inhibition of Th17 differentiation	IL-10, TGF-β, IDO	Down-regulated expression of RORγt, suppression of Th17 lineage commitment	[19]
Suppression of CTL-dependent toxicity	IL-10, TGF-β, IDO	Reduced CTL-dependent elimination of tumor cells	[18,19]
Pro-inflammatory reprogramming of tumor microenvironment	IFN-γ, IL-12, TNF-α	Increased TIL activation and cytolytic activity, Increased antigen presentation	[19]
Enhancement of T cell function under inflammatory stimuli	NO, ROS, type I IFNs	Suppression of regulatory cells, enhanced activation and cytotoxic function of CAR T cells	[23]
Enhanced recruitment of immune cells in tumor microenvironment	CCL5 (RANTES), CXCL9, CXCL10	Increased influx of CAR T cells into tumors	[23]
Formation of tertiary lymphoid structures	CCL19, CXCL13, TNF-α	Increased antigen presentation, enhanced CAR T cell priming	[24]
Support for survival, expansion, and cytotoxicity of CTLs	IFN-γ, IL-12, TNF-α	Enhanced persistence and killing-ability of CAR T cells	[24,25,26]
Protection of multiple myeloma cells from CAR T cells with lower affinity and moderate lytic activity	Survivin, Mcl-1	Inhibition of caspase-3/7 and suppression of Bim/Noxa pro-apoptotic axis in multiple myeloma cells, reduced cytotoxicity of low-affinity CAR T cells	[27,28]
Enhanced proliferation and survival of CAR T cells by engineered IL-7- and IL-12-overexpressing MSCs	IL-7, IL-12	Increased generation of IFN-γ and TNF-α-producing tumorotoxic T cells	[29,30]

## Data Availability

The data that are discussed in this article are presented in cited studies.
